# Sonophotocatalytic Dye Degradation Using rGO‐BiVO_4_ Composites

**DOI:** 10.1002/gch2.202100132

**Published:** 2022-03-02

**Authors:** Manish Kumar, M. N. M. Ansari, Imed Boukhris, M. S. Al‐Buriahi, Z. A. Alrowaili, Nada Alfryyan, P. Thomas, Rahul Vaish

**Affiliations:** ^1^ School of Engineering Indian Institute of Technology Mandi Mandi Himachal Pradesh 175005 India; ^2^ Institute of Power Engineering Universiti Tenaga Nasional Kajang Selangor 43000 Malaysia; ^3^ Department of Physics Faculty of Science King Khalid University P. O. Box 9004 Abha Saudi Arabia; ^4^ Laboratoire des matériaux composites céramiques et polymères (LaMaCoP) Département de Physique Faculté des Sciences de Sfax Université de Sfax BP 805 Sfax 3000 Tunisia; ^5^ Department of Physics Sakarya University Esentepe Campus Sakarya 54187 Turkey; ^6^ Physics department College of Science Jouf University P. O. Box 2014 Sakaka Saudi Arabia; ^7^ Department of Physics College of Science Princess Nourah bint Abdulrahman University P. O. Box 84428 Riyadh 11671 Saudi Arabia; ^8^ Central Power Research Institute Dielectric Materials Division Bengaluru Karnataka 560080 India

**Keywords:** BiVO _4_, composite, dyes, reduced graphene oxide, sonophotocatalysis

## Abstract

Reduced graphene oxide (rGO)/bismuth vanadate BiVO_4_ composites are fabricated with varied rGO amounts (0, 1, 2, and 3 wt%) through the synergetic effects of ultrasonication, photoinduced reduction, and hydrothermal methods, and the materials are tested as tools for sonophotocatalytic methylene blue (MB) dye degradation. The effect of rGO content on the sonophotocatalytic dye degradation capabilities of the composites are explored. Characterization of the proposed materials is done through transmission electron microscopy (TEM), X‐ray diffraction (XRD), X‐ray photoelectron spectroscopy (XPS), Fourier transformation infrared spectroscopy as well as scanning electron microscopy. The coexistence of BiVO_4_ and rGO is confirmed using Raman spectroscopy and XRD. TEM confirms the existence of interfaces between rGO and BiVO_4_ and XPS affirms the existence of varied elemental oxidation states. In order to investigate the charge carriers transportation, time‐dependent photocurrent responses of BiVO_4_ and 2 wt%‐ rGO/BiVO_4_ are done under visible light irradiation. The sonophotocatalytic MB dye degradation in an aqueous medium displays promising enhancement with rGO doping in rGO/BiVO_4_ composite. The 2 wt%‐ rGO/BiVO_4_ sample exhibits ≈52% MB dye degradation efficiency as compared to pure BiVO4 (≈25%) in 180 min of the sonophotocatalysis experiment. Phytotoxicity analysis through germination index is done using vigna radiata seeds.

## Graphical abstract

rGO/BiVO_4_ composites are found to be effective catalysts under sono‐photocatalysis. Photocurrent measurements confirm that thanks to the fabrication of this composite, rGO exibits enhanced photosensitivity. The composite shows promise as a water purification material.

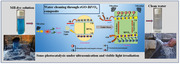

## 1 Introduction

Discharge of organic dyes as effluents from the printing and textile industries contaminate the water resources. To eradicate these pollutants from wastewater, semiconductor photocatalysis and piezocatalysis are most opted owing to their sustainable, low cost, and environment‐friendly approach.^[^ ^1^, ^2^ ^]^ For a few years, semiconductor materials have caught immense usage owing to their applications in water splitting, hydrogen production, organic dye degradation, and environmental remediation.^[^ ^3^ ^]^ Photocatalysis utilizes light energy for the generation and separation of electron–hole pairs which further undergo redox reaction for organic dye degradation.^[^ ^4^ ^]^ Semiconductor materials such as ZnO, ZnS, SnO_2_, CdS, SrTiO_3_, and CuO, are commonly used for photocatalytic dye degradation.^[^ ^5^, ^6^, ^7^, ^8^ ^]^ The fast recombination pace of the photogenerated charge carriers is considered to be a drawback in photocatalysis. Though photocatalysis is widely used to clean dye from wastewater, its lack of activity in dark conditions and little utilization of light energy urged the researchers to search for an alternative.^[^ ^9^, ^10^ ^]^ In piezocatalysis, vibrational energy is utilized to produce piezoelectric potential in piezoelectric materials. Electron–hole pairs generated during the piezocatalysis get separated easily in the effect of a piezoelectric field which additionally reacts with oxygen and water to form active species.^[^ ^10^, ^11^, ^12^ ^]^ Materials such as BaTiO_3_,^[^ ^13^ ^]^ MoSe_2_,^[^ ^14^ ^]^ and MoS_2_,^[^ ^15^ ^]^ have been used for piezocatalytic dye degradation.^[^ ^16^ ^]^ In view to discard the rapid recombination rate of the photogenerated charge carriers, piezoelectric potential has been introduced into photocatalysis.^[^ ^17^, ^18^ ^]^ The generated electric field due to polarization gives the driving force for transportation of the photogenerated charge carriers resulting in their separation.^[^ ^8^ ^]^ Thus organic dye degradation efficiency could be improved further through the combined effect of piezophotocatalysis.^[^ ^19^ ^]^ BiVO_4_ has been used as a photocatalyst owing to its narrow bandgap (2.4–2.5 eV), less toxicity, and low cost.^[^ ^20^, ^21^ ^]^ Monoclinic bismuth vanadate (m‐BiVO_4_) possesses fascinating properties such as solar energy absorption, ferroelasticity, ionic conductivity, hydrogen production, coloristic,^[^ ^22^ ^]^ environment‐friendliness, and chemical stability.^[^ ^3^, ^22^, ^23^ ^]^ The photocatalytic efficiency of m‐BiVO_4_ is confined because of its low charge transportation and fewer surface absorption phenomena.^[^ ^24^ ^]^ BiVO_4_ has a centrosymmetric structure and is not piezoresponsive.^[^ ^25^, ^26^ ^]^ Disrupted bonding on the surface and discontinuity in microstructure induces internal stress relaxation in BiVO_4_ imparting flexoelectric effect in the material.^[^ ^27^ ^]^ Because of the flexoelectric effect, centrosymmetric structures have been reported to show piezoresponsive behavior. During the formation of monoclinic fergusonite structure from tetragonal scheelite structure at a temperature below 255 °C, ferroelasticity phenomena get induced in BiVO_4_. Ferroelastic domains emerge over the surface to prevent the transformation, thus inducing piezoresponsive phenomena in BiVO_4_.^[^ ^25^, ^28^ ^]^ As BiVO_4_ is reported to show local surface piezoelectricity, piezocatalysis can be done on it.^[^ ^25^ ^]^ Using the piezoelectric force microscopy technique, Wei et al. affirmed the presence of piezoelectric phenomenon in Au/BiVO_4_.^[^ ^29^ ^]^

Reduced graphene oxide (rGO) possesses a 2D conjugated structure where an arrangement of carbon atoms constitutes a hexagonal honeycomb lattice.^[^ ^30^ ^]^ As graphene has extended π electron conjugation and high carrier mobility, it serves as an effective redox mediator which enhances the transportation of the photogenerated charges between the semiconductors.^[^ ^31^ ^]^ rGO has been known for its enhanced adsorption ability of varied inorganic and organic pollutants because of its hydrophobic, larger surface area, and stronger π–π interactions of its 2D aromatic structure.^[^ ^22^ ^]^ Thus, it would be an efficient way to increase the catalytic property of a material by coupling it with graphene‐related material (rGO). It is believed that interfaces of rGO/BiVO_4_ composite can establish a better charge separation phenomenon by enhancing the charge transfer rate.^[^ ^3^, ^32^, ^33^, ^34^ ^]^ Wang et al. showed enhanced photocatalytic efficiency by interfacial coupling of rGO/BiVO_4_.^[^ ^34^ ^]^ Photocatalytic dye degradation capability of rGO/BiVO_4_ composite has been already reported in the past studies.^[^ ^35^, ^36^ ^]^ Piezocatalytic dye degradation capability of rGO/BiVO_4_ composite has been reported by our research group recently.^[^ ^37^ ^]^ Dye degradation efficiency through combined sonophotocatalysis on rGO/BiVO_4_ has not been reported previously. So in this study, we aim to show the sonophotocatalytic performance of rGO/BiVO_4_.

## 2 Results and Discussion

**Figure 1:**
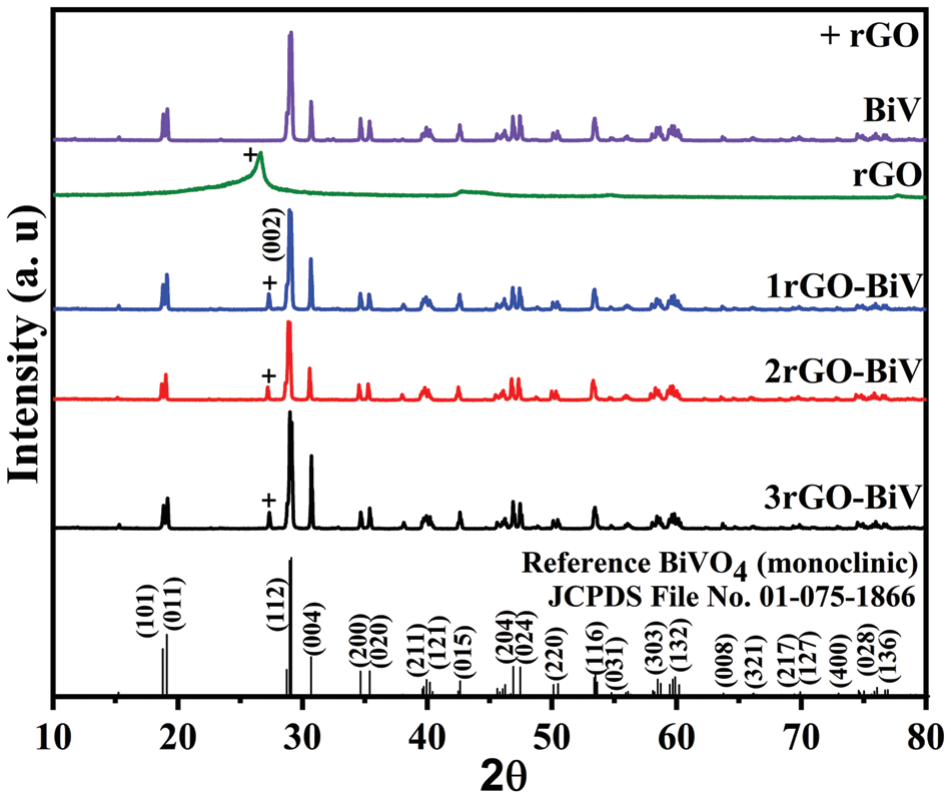
XRD plots of pure BiV, rGO, 1rGO‐BiV, 2rGO‐BiV, and 3rGO‐BiV samples.

**Figure** **1** shows X‐ray diffraction (XRD) diffraction plots of the BiV, rGO, 1rGO‐BiV, 2rGO‐BiV, and 3rGO‐BiV powders. The acquired sharp XRD peaks of all the samples show high crystalline quality. The peaks show well agreement with that of pure monoclinic phase BiVO_4_ in accordance with the standard Joint Committee on Powder Diffraction Standards 01‐075‐1866. All the peaks of rGO‐BiV depict similarity to that of the BiV except the presence of an additional peak at ≈27.2°, which affirms the formation of the monoclinic phase of BiVO_4_ in all other rGO‐BiVO_4_ samples.

The peak residing at ≈27.2° corresponds to the (002) plane of the rGO phase.^[^ ^38^ ^]^ The formation of the (002) plane in rGO‐BiV samples suggests the smooth incorporation of rGO by ultrasonic induced phenomena. Usually, at 26.5° position, the (002) plane of graphite is present and the peak of the (002) plane of rGO is found at 2θ angle less than that of graphite.^[^ ^39^ ^]^ Here we find the presence of rGO peak at 26.5° like that of graphite which may be caused due to intensified restacking of the rGO layers. Shift in peaks from 26.5° to 27.2° can be observed in all rGO‐BiV samples which may happen due to the intensified restacking phenomenon of rGO layers and/or additional rGO reduction due to the synergetic effects of ultrasonication, photoinduced reduction, and synthesis through the hydrothermal method^[^ ^39^, ^40^, ^41^ ^]^ Thus, XRD peaks with high crystallinity validate the presence of both BiVO_4_ and rGO in the fabricated composite.

**Figure 2:**
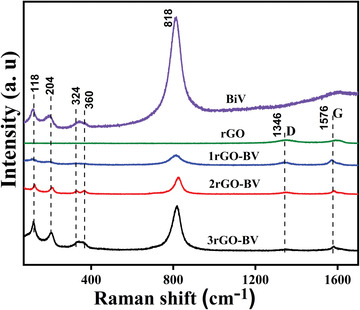
Raman spectra of the synthesized BiV, rGO, 1rGO‐BiV, 2rGO‐BiV, and 3rGO‐BiV powders.

In view to analyze the state of carbon‐based materials, Raman spectroscopy analysis was carried out. Raman spectra of BiV(BiVO_4_) and different compositions of rGO‐BiV are shown in **Figure** **2**. The presence of the BiVO_4_ phase in all the synthesized rGO‐BiV composites is affirmed by the Raman bands at near 818, 360, 324, 204, and 118 cm^–1^.^[^ ^21^ ^]^ Symmetric stretching of V—O is represented by the band at 818 cm^–1^. Symmetric and antisymmetric bending of V—O bonds is represented by the bands at 324 and 360 cm^–1^, respectively.^[^ ^42^ ^]^ Translation, as well as rotation species of the BiVO_4_ crystal lattice, is represented by the bands at 118 and 204 cm^–1^. In all rGO‐BiV composites, the bands at 1346 and 1576 cm^–1^ are attributed to rGO, which corresponds to G and D bands, respectively.^[^ ^22^ ^]^ The D band portrays symmetric hexagonal graphitic lattice interruption while the G band portrays in‐planar stretching of the symmetric sp^2^ C—C bond.^[^ ^43^ ^]^ In all rGO‐BiV composites, we observe a slight shift in D and G bands which may be due to the modification in surficial strain amid the interaction of BiVO_4_ and rGO.^[^ ^44^ ^]^

**Figure 3:**
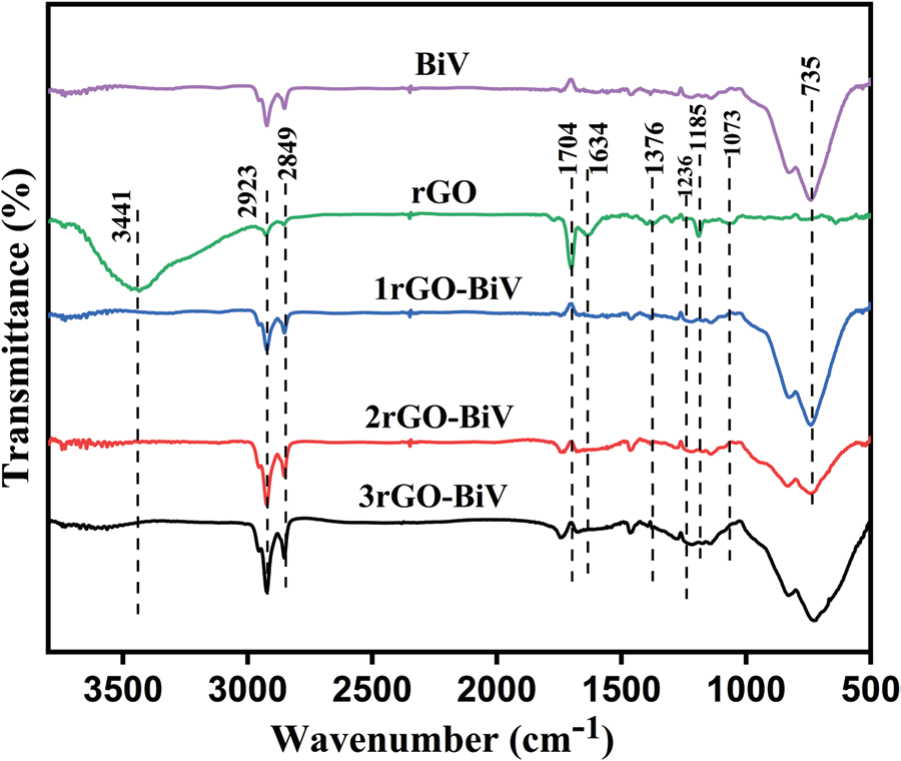
FTIR spectra of pure BiV, rGO, 1rGO‐BiV, 2rGO‐BiV, and 3rGO‐BiV samples.

In order to analyze the change in the functional group of the material Fourier transformation infrared (FTIR) spectroscopy was carried out. The FT‐IR spectra of the rGO, BiV, etc. have been shown in **Figure** **3**. The reduced graphene oxide spectrum shows the availability of oxygen containing groups. The peaks at 1704, 1236, and 1073 cm^–1^ depicts the C=O, C—O, and C=O=C vibrations, respectively. The presence of peak at 3441 cm^–1^ attributes to that of absorbed H_2_O and OH group of rGO. Symmetric and antisymmetric stretching vibration of the C—H bonds is represented by the peaks at 2923 and 2849 cm^–1^, respectively. Stretching vibration of the C=C is attributed to the peak at 1634 cm^–1^.^[^ ^45^ ^]^ The presence peak at 1376 cm^–1^ gives OH group bending vibration. The presence of peak at 1185 cm^–1^ depicts the stretching vibration of the C—C. Further the peak at 735 denotes symmetrical stretching vibration of VO_4_^3–^.^[^ ^46^, ^47^ ^]^

**Figure 4:**
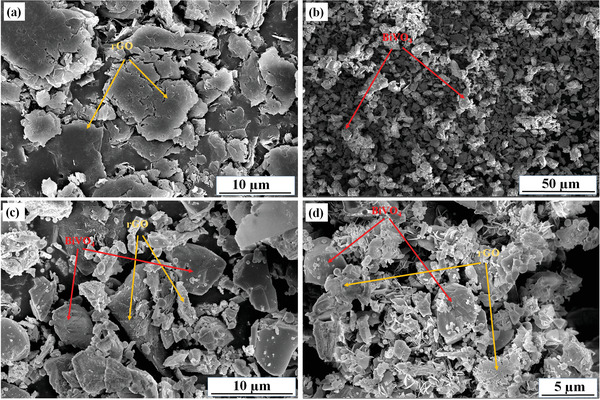
SEM images of a) rGO, b) BiV, c) 2rGO‐BiV, and d) 3rGO‐BiV.

The surface morphology was analyzed using the scanning electron microscopy (SEM) images of rGO, BiV, 2rGO‐BiV, and 3rGO‐BiV can be seen in **Figure** **4**a–d. A structured layer of rGO can be viewed in Figure 4a. BiVO_4_ particles show irregular morphology as in Figure 4b. As the rGO is incorporated in BiV, the combination influences nucleation and growth phenomenon of BiV on the rGO surface, thus enhancing the adhesion and dispersion of BiV on rGO sheets which further reduces the BiV particles agglomeration and restrains of rGO restacking.^[^ ^48^ ^]^ In 2rGO‐BiV and 3rGO‐BiV samples, we observe that BiVO_4_ particles are densely covered with the rGO nanosheets as shown in Figure 4c,d, which evince ample contact possibility and efficacious interfacial interaction amid BiVO_4_ and rGO. This close interaction endorses the transportation of electron from BiVO_4_ to rGO and thus provides efficacious charge separation within the composite material while sonophotocatalytic methylene blue (MB) dye degrade.

**Figure 5:**
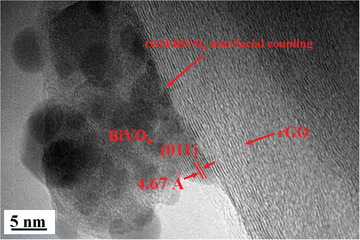
TEM image of 2rGO‐BiV depicting the formed interfacial coupling amid rGO and BiVO_4_.

The formation of interfacial coupling between BiVO_4_ and rGO was confirmed using transmission electron microscopy (TEM) images. The TEM image of the 2rG‐BiV composite shown in **Figure** **5** reveals the formation of compact interfacial coupling between the rGO and BiVO_4_. Fringe spacing *d* = 4.67 Å corresponding to BiVO_4_ crystallographic planes (011) can be observed.^[^ ^26^ ^]^ Thus, it can be evidenced that there is proper formation of interfacial coupling in between the rGO and BiVO_4_ phases in the composite sample.

**Figure 6:**
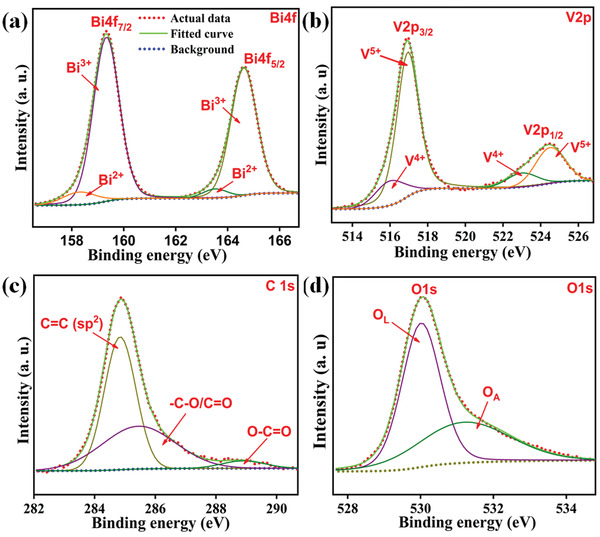
The XPS spectra of 2rGO‐BiV sample: a) Bi4f, b) V2p, c) C 1s, and d) O 1s.

The X‐ray photoelectron spectroscopy (XPS) spectrum of the 2rGO‐BiV sample which corresponds to V2p, Bi4f, C 1s, and O1s scans is clearly shown in **Figure** **6**a–d. Considering the Bi4f spectrum, initially, the peaks show Bi4f_7/2_ and Bi4f_5/2_ components which were additionally deconvoluted into Bi^3+^ and Bi^2+^ components.^[^ ^49^ ^]^ The existence of the Bi^3+^ oxidation state is affirmed through the peaks present at 159.2 and 165 eV while the peaks that are seen at 158 and 163 eV affirm the availability of Bi^2+^ oxidation state in the 2rGO‐BiV sample. Considering the V2p spectrum, initially, the peaks show V2p_1/_2 and V2p3_/2_ peaks which were additionally deconvoluted into V^4+^ and V^5+^ components.^[^ ^50^ ^]^ Peaks at 517 and 524 eV represent V^5+^ oxidation state while the intense peaks at 516 and 523 eV affirm the existence of V^4+^ oxidation state. Considering the O1s spectrum, there resides an asymmetric O1s component which is additionally deconvoluted into O_L_ and O_A_ components. The peak at 530.01 eV represent the O_L_ component which is assigned to the lattice oxygen (O^2–^) while the peak at 531.20 eV represents the O_A_ component which is assigned to oxygen vacancies.^[^ ^49^, ^51^ ^]^ The reduction of V^5+^ to V^4+^ and Bi^3+^ to Bi^2+^ occurs due to the intrinsic defects induced at the time of thermal treatment (i.e., localized oxygen vacancies) where the additional charge is trapped as electron pairs. This causes the occurrence of signal peaks of Bi^2+^ and V^4+^ as well as that of Bi^3+^ and V^5+^ in XPS spectra. The presence of rGO can be detected through the C species chemical state.^[^ ^52^ ^]^ C1s peaks in the 2rG‐BV sample can be attributed to sp^2^ hybridized carbon (C—C) species (≈284.6 eV), oxygen containing functional groups (C—O, epoxy and hydroxyl) species (≈286.4 eV) and ((C—O, carboxyl) species (≈288.6 eV).^[^ ^53^ ^]^

**Figure 7:**
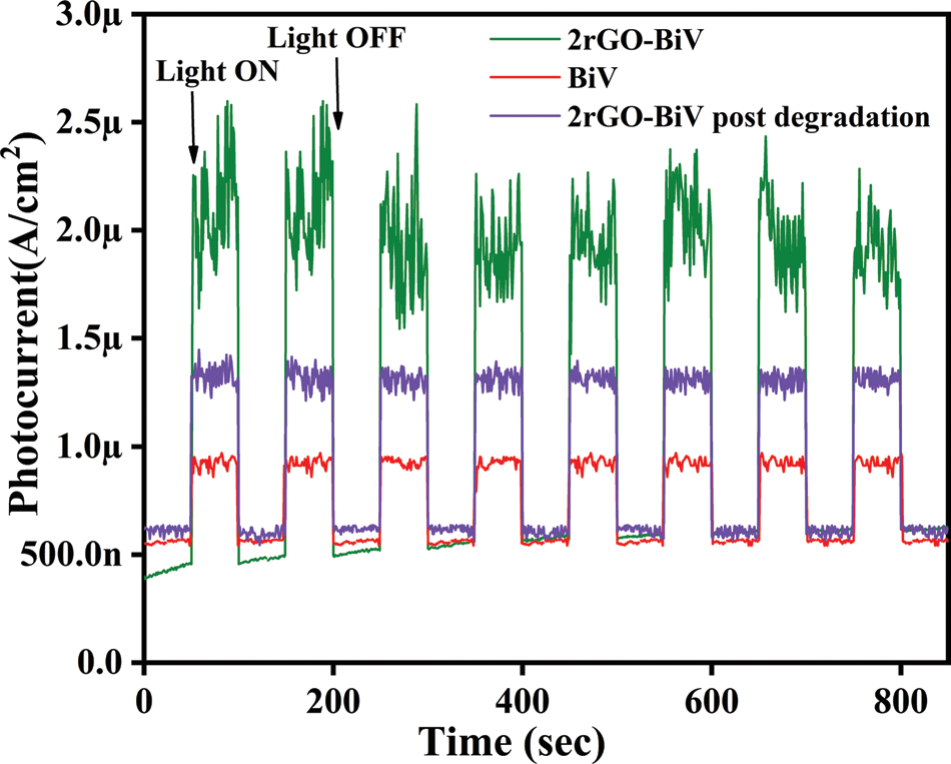
Current response versus time for BiV and 2rGO‐BiV before and after degradation in DI water.

In order to investigate the charge carriers transportation, time‐dependent photocurrent responses of BiV and 2‐rGO‐BiV were done under visible light irradiation. **Figure** **7** shows a low dark current density (≈500 nA cm^−2^) for BiV and 2rGO‐BiV before and post degradation. On illumination of visible light, BiV shows less than 1 µA photocurrent density while 2rGO‐BiV shows ≈2.5 µA photocurrent density before dye degradation which recedes to ≈1.4 µA photocurrent post degradation, thereby giving 2.5 times higher photocurrent density by 2rGO‐BiV than pure BiV sample. Thus, this enhanced photocurrent density can be attributed to lower recombination rate of the photogenerated electron–hole pairs. This suggests that rGO‐BiVO_4_ interfacial coupling reduces the photogenerated charge recombination rate. Thus rGO enhances catalytic activity by suppressing the photogenerated charge recombination. The photocurrent density response for the materials are reversible, so photocurrent can be reproduced for each irradiation.^[^ ^54^, ^55^ ^]^ Photocurrent given by 2rGO‐BiV recedes in value post dye degradation which suggests that there may have incurred some carbon losses owing to ultrasonication process involved. This could be considered as a future work to minimize carbon losses.

**Figure 8:**
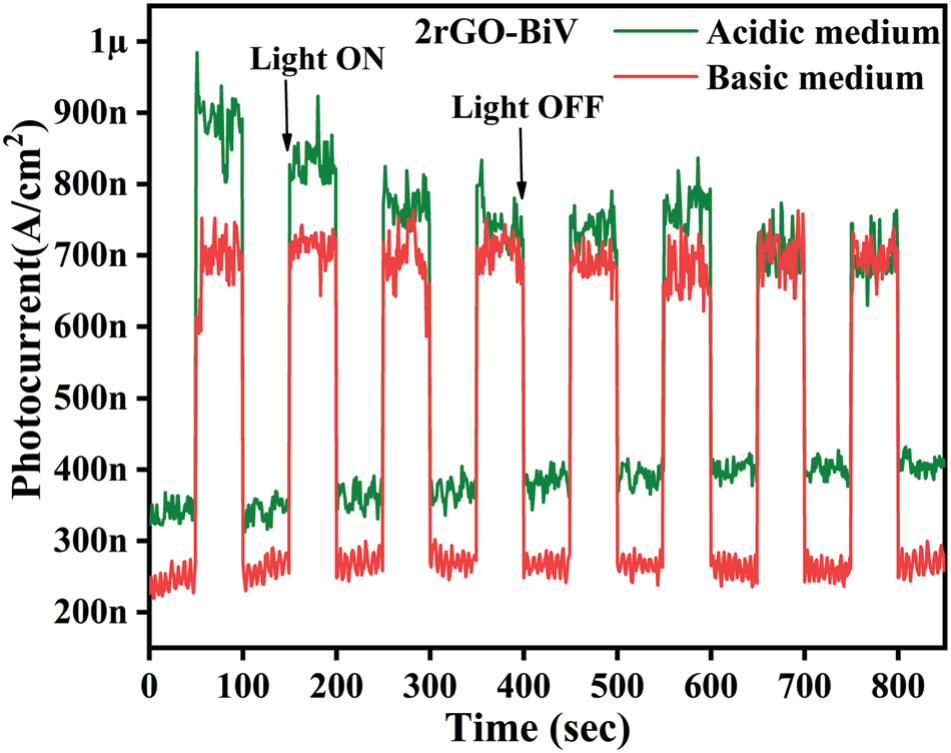
Current response versus time for 2rGO‐BiV in acidic and basic medium.

A chemical stability test of the best sample (2rGO‐BiV) was done by immersing the catalyst in acidic and basic solutions. 1 m hydrochloric acid solution in distilled water (DI) water was made for preparing acidic aqueous solution while for making basic solution 1 m NaOH has been used. Once the acidic and basic solution is prepared, 0.1 g of 2rGO‐BiV powder was added to each vial containing 10 mL of acidic and basic solution, respectively. Stirring was induced at 500 rpm for a duration of 24 h. After 24 h, the sample was retrieved back through centrifuge and then dried in oven at 80 °C for 24 h. After proper drying, the retrieved sample weight was found to be equivalent to the initial weight of the sample taken. This confers that the catalyst has mass stability in both acidic and basic aqueous solution even after 24 h. Photocurrent of the obtained sample were taken and the obtained results are shown in **Figure** **8**. It can be seen that low dark current density of ≈200 and ≈300 nA and on visible light illumination it rise to ≈700 and ≈900 nA for base and acid catalyst, respectively. Though there is decrease in current density but rise of current from dark to visible light is still nearly 2.5 times which is nearly same attained in DI water. The lower current density attained here may due to ambient parameters such as amount of coating on the sample but the increment in the current density is nearly same. Thus, there is mass stability and based on photocurrent result there may be chemical stability though it needs improvement in further study (Figure S1, Supporting Information).

**Figure 9:**
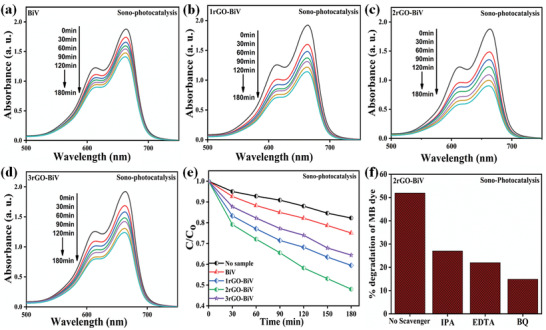
a–d) Absorption spectra obtained from the sonophotocatalytic experiment using BiV, 1rGO‐BiV, 2rGO‐BiV, and 3rGO‐BiV sample, b) $\frac{C}{C_{o}}$ versus time plots procured during sonophotocatalysis with all samples and without any sample, and c) effect of different scavengers on MB dye degradation percentage obtained from the sonophotocatalytic experiment using 2rGO‐BiV sample.

The sonophotocatalytic performance of rGO‐BiVO_4_ samples were investigated by the degradation of the MB dye and the procured results can be seen in **Figure** **9**a–f. Before the start of the sonophotocatalytic experiment, complete adsorption‐desorption saturation of MB dye was done for each sample and this was attained in the dark conditions with incessant stirring (500 rpm). The absorption spectra were procured while sonophotocatalytic experiment with the use of BiV, 1rGO‐BiV, 2rGO‐BiV, and 3rGO‐BiV sample is displayed in Figure 9a–d. Figure 9e shows the $\frac{C}{C_{o}}$ versus vibration time plots acquired from performing the sonophotocatalytic experiments devoid of and with the use of powdered samples. Under the synergetic effect of sonophotocatalysis, only 18% MB dye degradation was attained devoid of any sample usage. Local hot spots are formed due to the continuous rise and collapsion of water bubbles leading to a rise in localized temperature in the range of 4000–5000 K.^[^ ^56^ ^]^ Thermolytic decomposition of water leads to •OH radical formation which additionally causes MB dye degradation.^[^ ^56^ ^]^ This phenomenon is termed as “sonolysis.” BiV, 1rGO‐BiV, 2rGO‐BiV, and 3rGO‐BiV exhibited dye degradation efficiency of 25%, 40%, 52%, and 35% respectively within 180 min of sonophotocatalysis. The content of rGO in the composite has greatly influenced the dye degradation efficiency. Dye degradation efficiency showed an initial increment with an increase in the content of rGO up to 2 wt% followed by a decrease in activity with an additional increase in the content of rGO. The best sonophotocatalytic sample (2 wt%‐ rGO/BiVO_4_) attained ≈52% dye degradation in comparison to pure BiVO_4_ (≈25%). A comparison of piezophotocatalytic degradation rates using different catalysts is shown in **Table** **1**. Scavengers such as p‐benzoquinone (p‐BQ), ethylenediaminetetraactic acid, and isopropanol were utilized as scavengers for superoxide radical (•$O_{2}^{-}$), holes (h^+^), and hydroxyl radical (•OH), respectively. During the sonophotocatalytic experiment, these scavengers were separately added to the dye solution to trap the active species. The capture of the main active radical causes reduction in the sonophotocatalytic efficiency.^[^ ^57^, ^58^ ^]^ As per Figure 9f BQ scavenger which traps superoxide (•$O_{2}^{-}$) radicals put a drastic impact on the sonophotocatalytic activity. So we consider (•$O_{2}^{-}$) radicals as the major active radical. Major sonophotocatalytic efficiency is shown by 2rG‐BV sample among all fabricated samples. So, it can be deduced that with higher rGO wt% in the composite, BiVO_4_ particles get completely covered with the rGO sheets which result in the reduction of sonophotocatalytic efficiency.

**Table 1:** Piezophotocatalytic degradation rate using different catalyst

Catalyst	Model dye	Energy source (U‐ultrasonic; L‐light)	Catalyst form	Initial dye concentration (volume used)	Reaction time [min]	% Degradation
CuS/ZnO^[^ ^82^ ^]^	Methylene blue (MB) dye	U: 200 W; L: Xe lamp	Nanowires	5 mg L^−1^ (50 mL)	20	100%
Zn_1−_ *_x_* SnO_3_^[^ ^83^ ^]^	Methylene blue (MB) dye	U: 0.2 W, 40 kHz; L: UV light	Nanowires	4 ppm (10 mL)	120	75%
Ba_0.875_Ca_0.125_Ti_0.95_Sn_0.05_O_3_^[^ ^84^ ^]^	Methylene blue (MB) dye	U:–; L: UV light	Powder	5 mg L^−1^ (10 mL)	150	98%
ZnO^[^ ^85^ ^]^	Methylene blue (MB) dye	U: 1 Hz; L: UV light	Nanowires	5 mg L^−1^ (100 mL)	120	96%
KNbO_3_ nanostructures^[^ ^86^ ^]^	Rhodamine B (RhB)	U: 110 W, 40 kHz; L: Xe lamp	Powder	10 mg L^−1^ (100 mL)	120	92.6%
BaZr_0.02_Ti_0.98_O_3_^[^ ^87^ ^]^	Rhodamine B (RB dye)	U: 70 W, 40 kHz; L: UV light	Powder	6 mg L^−1^ (10 mL)	240	89%
BaTiO3 nanowires^[^ ^88^ ^]^	MO dye	U: 180 W, 40 kHz; L:UV light	Nanowires	5 mg L^−1^ (100 mL)	80	≈98.1%
BiOBr^[^ ^72^ ^]^	Rhodamine B (RB dye)	U: 120 W, 40 kHz; L: visible light	Powder	10 mg L^−1^ (50 mL)	120	100%
rGO/BiVO_4_ (present study)	Methylene blue (MB) dye	U: 150 W, 40 kHz; L: visible light	Powder	10 mg L^−1^ (10 mL)	180	≈52%
Na_0.5_Bi_0.5_TiO_3_^[^ ^89^ ^]^	Rhodamine B (RB dye)	U: 120 W, 40 kHz; L: visible light	Powder	10 mg L^−1^ (50 mL)	180	98%

**Figure 10:**
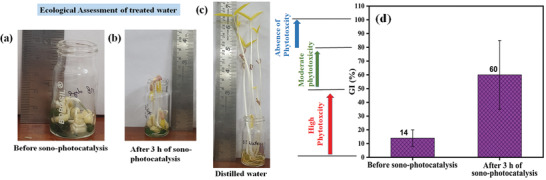
The effect of Mb dye on the growth of Vigna radiata seeds analyzed for 7 d. Treatment is done using a) 10 mg L^−1^ MB dye, b) treated wastewater, c) distilled water, and d) Germination index conducted on two samples post 0 and 3 h of sonophotocatalysis.

The germination index test was done with three vials with each having ten seeds of vigna radiata placed, where 0.5 mL of treated, untreated, and distilled water was poured each day. The test was conducted in the Indian Institutes of Technology Mandi, India at an environmental temperature of 25 °C. The analysis was carried for 7 d. **Figure** **10**a–c shows the growth of the seeds using dye water before sonophotocatalysis, treated water after 3 h of sonophotocatalysis, and distilled water. It was observed that the untreated 10 mg L^−1^ dye showed the most inhibition to the growth of the seeds when compared to the treated and control sample. The growth in the case of treated wastewater justifies a nontoxic approach toward the plant growth.^[^ ^59^ ^]^ However, the adverse effect still needs to be reduced and proper safety needs to be taken in the case of edible plants. We believe that this practice could reduce the load on natural water by making use of treated wastewater for the irrigation of parks and playgrounds.^[^ ^60^ ^]^ Figure 10d displays the results of the phytotoxicity. For the analysis of compounds based on Germination Index (GI) values, three classification categories have been proposed (1) high phytotoxicity(GI < 50%), moderate phytotoxicity 50% < GI < 80%, and an absence of phytotoxicity (GI > 80%). The toxicity level classification of dyes was followed as proposed by Emino et al.^[^ ^61^ ^]^ and Zucconi et al.^[^ ^62^ ^]^ The results show that the untreated dye water falls in high toxicity while the sonophotocatalytic treated water falls in the moderate phytotoxicity level.^[^ ^62^, ^63^ ^]^ It is to be taken into consideration that we could achieve ≈52% dye degradation efficiency only after the sonophotocatalytic experiment as per the UV spectroscopy results and we have used them for the germination index test. Hence, the germination index could be improved by attaining 100% cleaning of the wastewater through multiple ways such as increasing the catalytic time duration, increasing catalytic loading and decreasing the dye concentration.^[^ ^64^, ^65^ ^]^

**Figure 11:**
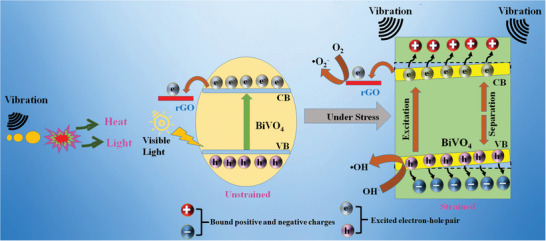
Sonophotocatalytic reaction mechanism for the rGO‐BiV composite.

A reaction mechanism for the rGO‐BiV composite for sonophotocatalytic MB dye degradation is displayed schematically in **Figure** **11**. Under visible light conditions, there incurs an electron–hole pair generation on the BiVO_4_ surface. The electrons from the valence band (VB) get excited to reach the conduction band leaving behind holes in the VB.^[^ ^66^ ^]^

The photogenerated electrons get transferred to the rGO sheets. In literature, BiVO_4_ exhibits localized surface piezoelectricity.^[^ ^25^ ^]^ As ultrasonication vibration is given, the localized surface piezoelectricity induces an internal electric field (polarization charges) in BiVO_4_.^[^ ^67^ ^]^ Polarization makes the holes and electrons move in the opposite direction and thus hinders the charge recombination possibility as the distance between the charge carriers is increased.^[^ ^64^, ^68^, ^69^ ^]^ Furthermore, the piezoelectric polarization field causes band bending phenomenon which helps in easy transportation of the charge carriers to the adsorbed pollutants.^[^ ^18^, ^69^ ^]^ Works of the literature suggests that the occurrence of defects at room temperature induces free charge carriers in piezoelectric materials.^[^ ^70^, ^71^ ^]^ There is still discussion in progress on the reason for the rise of free charge carriers during the ultrasonication process. However, according to literature, it may arise from the rise in thermal temperature due to the formation of local hot spots and sonoluminescence (wavelength less than 375 nm, light may form because of ultrasonication irradiation).^[^ ^72^, ^73^ ^]^ Local hot spots cause cavitation which in turn produces local strain.^[^ ^72^ ^]^ Sonocatalysis may be the reason behind the piezocatalytic effect in BiVO_4_. It is known that the nonpiezoelectric materials can degrade dye due to the sonocatalysis effect.^[^ ^74^ ^]^ In the synergetic effect when visible light is introduced in the sonocatalysis, there is a surge in free charge carriers in the catalyst. Thus, more electrons and holes reach the BiVO_4_ surface.^[^ ^75^ ^]^ Table 1 shows the degradation rate using different catalyst compared to the present study.

The free electrons generated in BiVO_4_ get transferred to the rGO sheets. Several factors assist in the charge transfer from BiVO_4_ to rGO sheets. It is suggested that when interjunction is formed between the n‐type BiVO4 and rGO, there is a flow of electrons from the BiVO_4_ surface to the rGO sheets till the Fermi level balances. Before contact, the work function of rGO sheets (4.75 eV) is less than that of BiVO_4_ (5.27 eV) while rGO holds a higher Fermi level than that of BiVO_4_. This causes band bending at the contact which in turn assists in the transportation of electrons from BiVO_4_ to rGO sheets.^[^ ^76^, ^77^ ^]^ Further, the high conductivity of the rGO and delocalized electrons present on the π–π graphitic carbon network also causes electron transfer.^[^ ^78^ ^]^ So, these free charge carriers get ambient time to participate in the catalytic surface reactions to form radicals. In sonophotocatalytic process, the electrons on the rGO sheets react with the adsorbed oxygen (O_2_) to form superoxide radicals ($O_{2}^{•-}$) and the holes present in the VB of BiVO_4_ react with the adsorbed water (H_2_O) to form the hydroxyl radicals (OH^•^)^[^ ^79^, ^80^ ^]^ These reactive oxygen species degrade the MB dye present in the water to make it harmless.

## 3 Conclusions

The rGO (BiVO_4_) composite material was fabricated for sonophotocatalytic MB dye degradation through the synergetic effects of ultrasonication, photoinduced reduction, and hydrothermal method. The sonophotocatalytic dye degradation efficiency showed an initial increment with an increase in the content of rGO up to 2 wt% followed by a decrement in activity with an additional increase in rGO content. The synthesized 2 wt% rGO‐BiVO_4_ composites depicted better sonophotocatalytic efficiency compared to all other composites which in turn could be attributed to the better interfacial coupling between the rGO and BiVO_4_ which helped in better charge transportation phenomenon. This has been further validated through time‐dependent photocurrent responses of BiV and 2‐rGO‐BiV under visible light irradiation.

## 4 Experimental Section

### Fabrication of BiVO_4_ Powder

BiVO_4_ was prepared through the use of Bi_2_O_3_ and V_2_O_5_ oxide powders. The stoichiometric molar ratio of these oxides was taken in an agate mortar, which was further mixed and ground manually for nearly 30 min. The retrieved fine powder mixture was put in a furnace for calcination at 700 °C for a duration of 8 h for the BiVO_4_ (BiV) phase formation. The retrieved powdered sample was then ball‐milled for 12 h at 250 rpm.

### Fabrication of rGO‐BiV

**Scheme 1:**
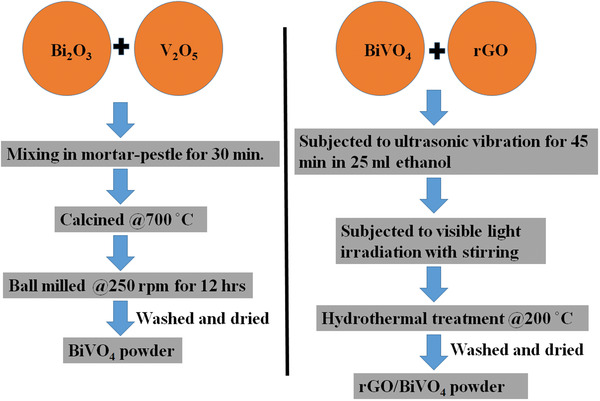
Pictorial illustration showing the synthesis method of rGO‐BiV.

The composites of rGO/BiVO_4_ were prepared through the visible light irradiated photocatalytic induced rGO using BiVO_4_ as shown in **Scheme** **1**. Reduce Graphene oxide was purchased from Ad‐Nano RGO, ADRGO, India. The quantity (1 , 2, and 3 wt%) of rGO was disseminated in ethanol solution (25 mL) in individual vials thereby the remaining wt% of BiVO_4_ sample were added to each vial. The vials were subjected to ultrasonication for 45 min at 30 °C till a homogenous solution is obtained. Then, with continuous stirring, the mixture was illuminated by three Havells bulbs (15 W power each) for 48 h. Stirring induces enhanced reduction of the rGO through the photocatalytic activity of BiVO_4_. The obtained sample was then fetched in a stainless‐steel autoclave (Teflon lined, 100 mL capacity). The autoclave was filled with solution and subjected to heat treatment at 200 °C for 8 h after sealing it appropriately. Once the autoclave naturally cools to room temperature, the acquired precipitates were washed three times using distilled water and ethanol. The final material was dried at 70 °C for 4 h duration in the oven.

### Characterization of rGO‐BiV

X‐ray diffractometer (XRD) (Rigaku diffractometer, Japan made (9 kW Cu‐*Kα* anode)) was used to determine the structural analysis of the sample. Sample scan was done over 10–80 scan range at 3°/min scan rate. Raman spectroscopy (HORIBA, Model‐LabRAM HR Evolution) with 532 nm laser excitation was utilized to know the bonds present in all the samples. The scan range provided was 100–1800 cm^–1^. FTIR spectroscopy were measured through the Perkin Elmer Spectrum RX I spectrophotometer with KBr pellets as sample matrix in the range of 400–4000 cm^–1^. The morphology and microstructures of the sample were observed through Nova Nano SEM‐450 field emission scanning electron microscope. The absorption spectra of the samples were analyzed using UV–vis–NIR Spectrophotometer (Shimadzu UV‐3150a). TEM with the use of FP‐Tecnai G2 S‐TWIN TEM instrument (FEI made in USA) was utilized to depict the interfacial coupling existing between BiVO_4_ and rGO. An Al‐Kα source (Nexsa X‐ray photoelectron spectrophotometer) was utilized to obtain the XPS spectrum in view to get the chemical state and binding energy of the rGO/BiVO_4_ sample.

### Photocurrent Measurement

Current–time curves were obtained through electrochemical workstation (AUT86543 Metrohm Autolab B.V.), in the three electrodes setup using platinum wire as the counter electrode while Ag‐AgCl wire used as a reference electrode and a working electrode with use of two bulbs (Havells company bulbs (15 W each) as the excitation source of light. Several cycles of ON and OFF with visible light was performed to obtain the photocurrent response. Here 0.1 m phosphate‐buffered saline aqueous solution was prepared to be used as electrolyte. In order to prepare working electrode, homogenous catalyst ink solution was made using 5 mg of the prepared catalyst dispersed in 1 mL of ethanol along with 20 µL Nafion solution. This catalyst ink was ultrasonicated for 30 min duration to obtain homogenous ink. The catalyst ink (≈10 µL) was used to coat the precleaned glassy carbon electrode surface. After proper drying of the coating, the electrode was used as working electrode.

### Sonophotocatalytic Experiment

The sonophotocatalytic efficiency of the prepared rGO/BiVO_4_ sample was analyzed by degrading MB dye. MB dye degradation was done using 0.05 g of each sample under the synergetic activity of visible light condition and ultrasonic vibration imparted by two Havells company bulbs (15 W each) and ultrasonic cleaner (40 kHz, 150 W), respectively. Before the start of the sonophotocatalytic experiment, complete adsorption–desorption saturation of MB dye of each sample was attained in dark conditions with incessant stirring (500 rpm). On attainment of adsorption saturation, the prior taken‐in‐use dye was changed, and afresh 10 mL dye of ≈10 mg L^−1^ concentration was utilized. Since water is being used as a medium in the ultrasonicator, so to discard the possibility of dye heating, the water medium was replaced after every 15 min interval. Dye solution of 1 mL was acquired after every 30 min interval of the sonophotocatalysis experiment which was later fetched back to the beaker to maintain a constant volume. Equation (1) is utilized to know the dye removal percentage.^[^ ^81^ ^]^

(1) $$\begin{array}{c} \%\;\text{Removal}\;\text{of}\;\text{MB}\;\text{dye}=\frac{C_{o}-C}{C_{o}}\times 100 \end{array}$$

where the symbol *C*_o_ and *C* signify the initial dye concentration and that after time “*t*,” respectively.

### Phytotoxicity Test

Root length and seed germination tests were conducted on distilled water and on two samples post 0 and 3 h of sonophotocatalysis. A simple test to understand the sustainability and suitability of the degraded MB dye water collected after sonophotocatalysis was conducted to see the germination of the Vigna radiata seeds. This test was done to understand the possibility of the reusage of wastewater. The test was done with three vials with each having ten seeds of vigna radiata placed, where 0.5 mL of treated, untreated, and distilled water was poured each day. The test was conducted in Mandi, India at an environmental temperature of 25 °C. The total count of germinated seeds along with their root length was noted as per AFNOR ISO 17126.^[^ ^59^ ^]^ The analysis was done for 7 d duration. The calculation of the germination index was done as per the formula in Equation (2).^[^ ^63^ ^]^

(2) $$\begin{array}{c} \text{GI}\;\left(\%\right)=\frac{\text{Seed}\;\mathrm{germination}\;\left(\%\right)\times \text{root}\;\text{length}\;\text{of}\;\text{treatment}}{\text{Seed}\;\mathrm{germination}\;\left(\%\right)\times \text{root}\;\text{length}\;\text{of}\;\text{control}}\;\times 100\; \end{array}$$

## Conflict of Interest

The authors declare no conflict of interest.

## Supporting information

Supporting Information

## Data Availability

The data that support the findings of this study are available from the corresponding author upon reasonable request.
