# Supplementary material for: Sonophotocatalytic Dye Degradation Using rGO‐BiVO4 Composites
Source: Glob Chall. 2022 Mar 2;6(6):2100132. doi: 10.1002/gch2.202100132 (PMC9189135; doi:10.1002/gch2.202100132)
Supplement: Supplementary file 1 — Supporting Information [file GCH2-6-2100132-s001.pdf]

Global Challenges
Open Access

Supporting Information

for *Global Challenges,* DOI: 10.1002/gch2.202100132

Sonophotocatalytic Dye Degradation Using rGO-$BiVO_4$ Composites

Manish Kumar, M. N. M. Ansari, Imed Boukhris, M. S. Al-Buriahi, Z. A. Alrowaili, Nada Alfryyan, P. Thomas, and Rahul Vaish**

As seen in Fig. S1. we even tested for the reusability with higher concentration dye which shows that with 20 mg/L dye concentration attains ~52 % degradation in 6 hrs which is same as that obtained with 10 mg/L in 3 hrs duration while in 3 hrs 20 mg/L dye attains ~32% degradation during sono-photocatalysis.

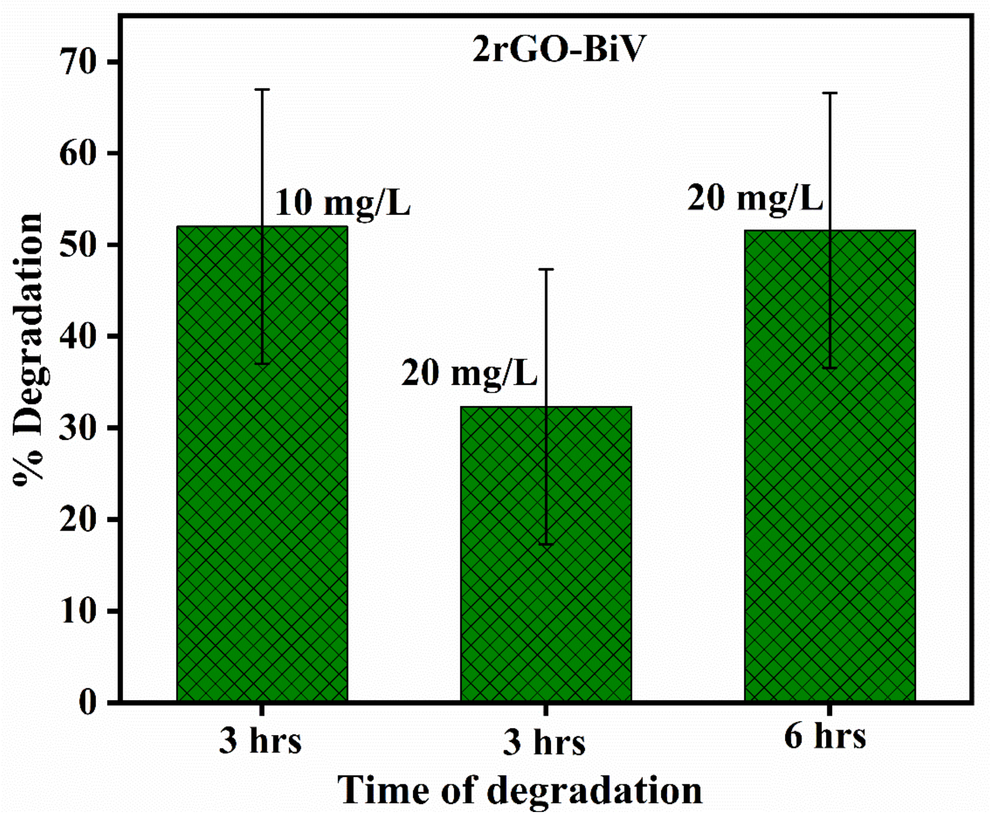

Fig. S1. Sono-photocatalytic degradation of MB dye using 2rGO-BiV sample.
